# Cognitive performance under stress: An experimental study in fire cadets

**DOI:** 10.1192/j.eurpsy.2021.2065

**Published:** 2021-08-13

**Authors:** N. Lebedeva, A. Balakaeva

**Affiliations:** 1 Diagnostics Department, Moscow Metropolitan Governance University, Moscow, Russian Federation; 2 Faculty Of Psychology, Lomonosov Moscow State University, Moscow, Russian Federation

**Keywords:** cognitive performance, cognitive reflection test, stress

## Abstract

**Introduction:**

Future firefighters are trained and selected for a job requiring the ability to perform well under high stress and time pressure.

**Objectives:**

The research is focused on the experimental study of fire cadets’ cognitive performance indicators (speed/accuracy) under stress.

**Methods:**

The study follows Solomon Four Group Design with two variables: stress stimuli (exposure/non-exposure) and participants’ background (50 male fire cadets; 50 male civilian students). Stress stimuli consisted of emergencies’ photos, audio, videos. ECG, EMG, systolic wave amplitude, pulse transit time were measured during the experiment to determine the respondents’ stress levels. The cognitive reflection test (CRT) was performed. Mann-Whitney U-test, Spearman’s rank correlation coefficient were used.

**Results:**

There were no differences between students and fire cadets in CRT time (p=0.515, students: 118.1±38.6 sec, cadets: 143.5±78.1 sec) and accuracy (p=0.246, students: 1.2±0.9, cadets: 1.4±0.9). Fire cadets in the stress exposure group (mean time=122, mean accuracy=1.22) performed CRT significantly faster (p=0.039) than non-exposed cadets (mean time=166, mean accuracy=1.56). The accuracy difference was insignificant (p=0.206). Fire cadets with prior emergency work experience (n=30, mean time=159.7, mean accuracy=1.6) were no different from other cadets (n=20, mean time=159.7, mean accuracy=1.1) both in time (p=0.289) and accuracy(p=0.07). The performance difference between civilian student groups was insignificant (exposure: mean time=123, mean accuracy=1.32; non-exposure: mean time=113, mean accuracy=1.06).

**Conclusions:**

Stress exposure enhances fire cadets’ CRT performance (in speed, but not in accuracy). Emergency work experience did not contribute to this effect, which could be explained by the self-selection effect (since only people inclined to emergency work choose to become a firefighter).

**Disclosure:**

No significant relationships.

